# Serum Resistin and Glomerular Filtration Rate in Patients with Type 2 Diabetes

**DOI:** 10.1371/journal.pone.0119529

**Published:** 2015-03-26

**Authors:** Lorena Ortega Moreno, Lucia Salvemini, Christine Mendonca, Massimiliano Copetti, Concetta De Bonis, Salvatore De Cosmo, Alessandro Doria, Vincenzo Trischitta, Claudia Menzaghi

**Affiliations:** 1 Research Unit of Diabetes and Endocrine Diseases, IRCCS “Casa Sollievo della Sofferenza”, San Giovanni Rotondo, Italy; 2 Research Division, Joslin Diabetes Center, Boston, MA, United States of America; 3 Unit of Biostatistics, IRCCS “Casa Sollievo della Sofferenza”, San Giovanni Rotondo, Italy; 4 Department of Medical Sciences, Division of Internal Medicine, IRCCS “Casa Sollievo della Sofferenza”, San Giovanni Rotondo, Italy; 5 Department of Medicine, Harvard Medical School, Boston, MA, United States of America; 6 Department of Experimental Medicine, Sapienza University of Rome, Rome, Italy; University of Verona, Ospedale Civile Maggiore, ITALY

## Abstract

**Background:**

High serum levels of the pro-inflammatory adipokine resistin have been associated with decreased renal function in the general population. The goal of this study was to investigate whether such association is also present among diabetic subjects, who are at increased risk of renal function loss.

**Methods:**

The cross-sectional association between serum resistin levels and estimated glomerular filtration rate (eGFR) was investigated in 1,560 type 2 diabetic (T2D) patients of European ancestry comprised in two different cohorts: 762 patients from San Giovanni Rotondo (SGR; Italy) and 798 patients from Boston (US).

**Results:**

Serum resistin was inversely associated with eGFR in SGR [β (SE) for one SD of resistin increment = -1.01 (0.70) ml/min/1.73m^2^, p = 0.019] and in Boston [β (SE) **= -**5.31 (0.74) ml/min/1.73m^2^, p < 0.001] samples, as well as in the two studies combined [β (SE) **= -**3.42 (0.52) ml/min/1.73m^2^, p < 0.001]. The association was unaffected by adjustment for smoking habits, BMI, waist circumference, diabetes duration, HbA1c, insulin treatment, hypertension and lipid-lowering therapy: β (SE) for one SD of resistin increment = -1.07 (0.70), p = 0.02; -5.50 (0.88), p < 0.001; and -2.81 (0.55) ml/min/1.73m^2^, p < .001, in SGR, Boston and the two studies combined, respectively. The association was significantly stronger in men than in women (p for resistin-by-gender interaction = 0.003). For each resistin SD increment, the odds of having eGFR < 0 ml/min/1.73m^2^ increased by 22% (OR = 1.22; 95% CI 1.02–1.44; p = 0.025) in SGR sample, 69% (OR = 1.69; 95% CI 1.38–2.07; p < 0.001) in Boston sample, and 47% (OR = 1.47; 95% CI 1.29–1.68; p < 0.001) in the two studies considered together. Similar associations were observed in the adjusted model: OR 95% CI for each SD resistin increment being 1.23 (1.03–1.46), p = 0.021; 1.52 (1.20–1.92), p < 0.001; 1.33 (1.16–1.53), p < 0.001, in SGR, Boston and the two studies combined, respectively.

**Conclusions:**

This is the first report of an association between high serum resistin and low eGFR in patients with T2D of European ancestry.

## Introduction

Chronic kidney disease (CKD), defined as reduced glomerular filtration rate (GFR), is a leading cause of premature death in patients with type 2 diabetes (T2D) [1–3]. A better understanding of the pathogenic mechanisms responsible for GFR decline in these patients is urgently needed to devise new interventions aimed at tackling this problem.

Besides hyperglycemia, many metabolic features of T2D, such as obesity, hypertension, and dyslipidemia, all clustering in insulin resistance, are known to have deleterious effects on kidney function [4,5]. Chronic low-grade inflammatory—another characteristic features of the diabetic state—has also been recognized as an important pathogenetic factor [6,7].

Cytokines secreted by adipose tissue (collectively known as adipokines) have been linked to both insulin resistance and low-grade inflammation [8]. Among them is resistin—a 12.5 kDa cysteine-rich protein that in humans is primarily secreted by the macrophages [9,10] embedded in the adipose tissue—which has been implicated in cardiovascular disease and atherogenesis [11–13]. Several cross-sectional studies have reported an inverse relationship between serum resistin levels and kidney function [14–17]. Such association has been described in the general populations [16] and in selected clinical settings [14,15,17] but not in T2D, for which only scant data, derived from *post-hoc*, subgroup analyses, are available [15].

To gain insights into the role of resistin in kidney function in diabetes, we investigated whether increased resistin levels are associated with low eGFR in more than 1,500 T2D patients of European ancestry from two different geographical regions.

## Subjects and Methods

### The San Giovanni Rotondo sample (SGR)

Baseline values of 762 subjects with T2D (defined according to the ADA 2003 criteria) from Gargano (Southern-Centre Italy) were used for this study. They are part of the Gargano Mortality Study, a cohort of consecutively recruited diabetic patients used for prospective investigation on determinants of all-cause mortality in T2D. The general features of this study have been previously described [18,19].

### The Boston sample

Baseline values of 798 subjects with T2D (defined according to the ADA 2003 criteria) who lived in the greater Boston area and received treatment at the Joslin Clinic and/or the Beth Israel Deaconess Medical Center (BIDMC) at the time of their recruitment, were used for this study. They are part of the Joslin Hearth Study which is a case-control study, comprising diabetic patients with (i.e. cases) or without (i.e. controls) coronary artery disease. The general features of this study have been previously described [19,20].

### Data Collection and Definitions

Clinical data were obtained from a standardized interview and examination. Body mass index (BMI) was calculated by dividing the weight (in kilograms) by squared height (in meters). Hypertension was defined as a systolic blood pressure was > 130 mmHg or diastolic blood pressure was > 85 mmHg or presence of antihypertensive therapy. Smoking habits, dyslipidemia (as indicated by the presence of lipid-lowering therapy), and insulin treatment were also recorded at the time of examination. Data regarding medications were confirmed by review of medical records. Individuals who reported smoking cigarettes regularly during the year before the examination were considered current smokers. Diabetes duration was calculated from the current age and the age at diagnosis of diabetes.

In the SGR sample, blood samples were collected between 8:00 and 9:00 AM after an overnight fast. In the Boston sample, blood samples were obtained between 7:00 AM and 6:00 PM without the requirement of fasting. Serum aliquots were stored at -80°C.

Serum creatinine was measured by using the modified kinetic Jaffè reaction. Estimated GFR was then calculated by using both MDRD and CKD-EPI equations [21,22].

### Ethics

The study protocols and the informed consent procedures were approved by the Institutional Ethic Committee of Istituto di Ricovero e Cura a Carattere Scientifico (IRCCS) ‘‘Casa Sollievo della Sofferenza” and of the Joslin Committee on Human Studies and the Beth Israel Deaconess Medical Center Committee on Clinical Investigations, respectively. All participants gave written informed consent.

### Measurement of Circulating Resistin Levels

Serum resistin concentrations were measured by a commercial ELISA (Bio Vendor, Brno Czech Republic) at the Research Unit of Diabetes and Endocrine Diseases at ‘‘Casa Sollievo della Sofferenza”, as previously described [23]. Inter—and intra-assay coefficients of variation were 7.0–8.1% and 5.2–6.6%, respectively.

### Statistical Methods

Patients’ baseline characteristics are reported as mean ± standard deviation (SD) and percentages for continuous and categorical variables, respectively.

Baseline comparisons between groups were performed using Pearson χ^2^ test for categorical variables, T-test and ANOVA models for normal-distributed continuous variables and Mann-Whitney U test for skewed-distributed continuous variables.

The association between resistin levels and eGFR (i.e. the dependent variable) as a continuous trait was investigated by multivariate linear regression analysis after logarithm transformation. Covariates were smoking habits, BMI, waist circumference, diabetes duration, HbA1c, insulin treatment, hypertension and lipid-lowering therapy, chosen because of their known influence on GFR. Age was not used as covariate because it is already included in the MDRD formula and also because of its co-linearity with disease duration. Because of the intrinsic nature of its design (i.e. case-control), analyses of the Boston study were adjusted by coronary artery disease status (i.e. yes/no, which is 0/1).

Results were reported as linear model coefficients along with their standard errors [β (SE)]. The association between resistin levels and eGFR as a dichotomous trait (i.e. ≥ or <60 ml/min/1.73m^2^) was tested by multivariable logistic regression analysis. Results were reported as Odds Ratios (OR), along with their 95% confidence interval (95% CI).

Random effect meta-analysis was performed in an individual patient data meta-analysis fashion [24] after checking for heterogeneity (i.e. the presence of a significant exposure-by-sample interaction).

A p-value 0.05 was considered as significant. All analyses were performed using SPSS v.15 (SPSS, Chicago IL) and SAS Release 9.1.3 (SAS Institute, Cary, NC, USA).

## Results

Clinical features of patients of both studies are shown in Table 1 and Table A in S1 File.

**Table 1:** Clinical characteristics of patients from SGR and Boston studies.

	**SGR sample (n = 762)**	**Boston sample (n = 798)**
Sex (males %)	388 (50.9)	521 (65.3)
Age (yrs)	62.0±9.6	64.5±6.8
Smokers (%)	173 (22.7)	399 (50.0)
BMI (kg/m^2^)	30.9±5.5	32.2±5.7
Waist circumference (cm)	102.2±13.5	113.6±13.2
Diabetes duration (yrs)	10.9±9.1	12.8±7.8
HbA1c (%)	8.7±1.9	7.4±1.3
Insulin treatment (%)	318 (41.7)	365 (45.7)
Hypertension (%)	392 (51.4)	596 (74.7)
Lipid-lowering therapy (%)	255 (33.5)	608 (76.2)
Resistin (ng/ml)	10.2±8.2	7.5±5.4
eGFR (ml/min/1.73m^2^)	74.0±19.8	70.2±21.5

Continuous variables were reported as mean ± SD whereas categorical variables were reported as total frequency and percentages. SGR: San Giovanni Rotondo; BMI: Body Mass Index; HbA1c: glycated haemoglobin;, eGFR: estimated glomerular filtration rate.

The two samples were quite different in terms of most clinical variables (p < 0.05) but, age and anti-hyperglycemic treatment. Correlations between resistin circulating levels and clinical features are shown in Table B in S1 File. In both samples, as well as in the combined data, which were analyzed by random effect individual meta-analysis, because of the presence of heterogeneity across samples (p of heterogeneity = 3.3*10^−6^), an inverse association was found between serum resistin and eGFR, considered as a continuous trait: β (SE) for one SD of resistin increment = -1.01 (0.70), p = 0.019; -5.31 (0.74), p < 0.001 and -3.42 (0.52) ml/min/1.73m^2^, p < 0.001, in SGR, Boston and the two studies combined, respectively (Table 2).

**Table 2:** Association between serum resistin levels and eGFR (continuous trait).

	**SGR sample (N = 762)**		**Boston sample (N = 798)**		**Individual data meta-analysis (1,560)** *	
	**β (SE)**	**P**	**β (SE)**	**P**	**β (SE)**	**P**
**Model 1**	-1.01 (0.70)	0.019	-5.31 (0.74)	<0.001	-3.42 (0.52)	<0.001
**Model 2**	-1.07 (0.70)	0.02	-5.50 (0.88)	<0.001	-2.81 (0.55)	<0.001

SGR: San Giovanni Rotondo.

The β linear coefficients represent the change in eGFR level for 1SD increase in resistin. SE: standard error.

Model 1 = unadjusted (Boston sample was adjusted by coronary artery disease status-yes/no).

Model 2 = adjusted by smoking habits, BMI, waist circumference, diabetes duration, HbA1c, insulin treatment, hypertension and lipid-lowering therapy (Boston sample was adjusted by coronary artery disease status-yes/no).

*Since the effect in SGR was different than that in Boston sample (p for beta values heterogeneity being = 3.3*10^−6^), individual data meta-analysis was carried out by using random effects.

Results were essentially the same after adjustments for smoking habits, BMI, waist circumference, diabetes duration, HbA1c, insulin treatment, hypertension and lipid-lowering therapy in both samples as well as in the combined analysis: β (SE) for one SD of resistin increment = -1.07 (0.70), p = 0.02; -5.50 (0.88), p < 0.001; and -2.81 (0.55) ml/min/1.73m^2^, p < 0.001, respectively (Table 2). In this combined analysis, the association between resistin and eGFR was significantly different across sexes: adjusted β (SE) per one SD of resistin increment being -3.62 (0.76) and -1.62 (0.78) ml/min/1.73m^2^ in men (n = 909) and in women (n = 651), respectively (p for resistin-by-sex interaction = 0.003). In this pooled analysis, resistin levels were not different in men as compared to women (p = 0.50).

Very similar results were obtained in both SGR [adjusted β (SE) = -1.45 (0.63) ml/min/1.73m^2^, p = 0.005)] and Boston [adjusted β = -5.37 (0.82) ml/min/1.73m^2^, p < 0.001)] samples when CKD-EPI, rather than MDRD, formula was used for measuring eGFR.

In both studies, serum resistin concentrations were significantly higher in patients with eGFR < 60 ml/min/1.73m^2^ compared with patients with eGFR ≥ 60 ml/min/1.73m^2^ (p < 0.001); (Table A in S1 File). For each SD increment in resistin levels, the odds of eGFR < 60 ml/min/1.73m^2^ increased by 22% (OR = 1.22; 95% CI 1.02–1.44; p = 0.025) in SGR sample, 69% (OR = 1.69; 95% CI 1.38–2.07; p < 0.001) in Boston sample, and 47% (OR = 1.47; 95% CI 1.29–1.68; p < 0.001) in the two combined samples which were analyzed by random effect individual meta-analysis, because of the presence of heterogeneity across them (p of heterogeneity = 0.014) (Table 3).

**Table 3:** Association between serum resistin levels and low eGFR (<60ml/min/1.73m^2^).

	**SGR sample (N = 762)**		**Boston sample (N = 798)**		**Individual data meta-analysis (1,560)** *	
	**OR (95% CI)**	**p**	**OR (95% CI)**	**p**	**OR (95% CI)**	**p**
**Model 1**	1.22 (1.02–1.44)	0.025	1.69 (1.38–2.07)	<0.001	1.47 (1.29–1.68)	<0.001
**Model 2**	1.23 (1.03–1.46)	0.021	1.52 (1.20–1.92)	<0.001	1.33 (1.16–1.53)	<0.001

SGR: San Giovanni Rotondo;

OR (95% CI) are given for SD increase of resistin levels.

Model 1: unadjusted (Boston sample adjusted by coronary artery disease status-yes/no).

Model 2: adjusted by smoking habits, BMI, waist circumference, diabetes duration, HbA1c, insulin treatment, hypertension and lipid-lowering therapy (Boston sample adjusted by coronary artery disease status-yes/no).

*Since the effect in SGR was different than that in Boston sample (p for OR values heterogeneity being = 0.014), individual data meta-analysis was carried out by using random effects

These associations were unaffected by adjustment for the same covariates considered above: OR for each SD resistin increment being 1.23 (1.03–1.46), p = 0.021; 1.52 (1.20–1.92), p < 0.001 and 1.33 (1.16–1.53), p < 0.001, in SGR, Boston and the two studies combined, respectively (Table 3).

## Discussion

In this study of a large number of T2D patients of European ancestry, we have shown that resistin levels are inversely related to eGFR and that the proportion of individuals with eGFR < 60 ml/min/1.73m^2^ increases significantly with increasing resistin levels. The association between resistin and eGFR was observed in two populations from different geographical regions, supporting a broad generalizability of these data. To the best of our knowledge, our finding is the first indicating such association among patients with T2D and resembles data so far obtained in the general population [16] and specific clinical sets as well [14,15,17].

Of note, the association we observed was significantly stronger among men than women. A similar sexual dimorphism has been previously reported for the association between adiponectin and chronic renal disease [25] or cardiovascular mortality [26]. Whether the sex specific association we observed is due to interaction between resistin and either sex-linked genes and/or sexual hormone effects is not known and deserves future investigations. Nonetheless, while waiting for further studies in additional samples, our finding definitively suggests the need of taking into account possible sex specific associations when addressing the role of pro-atherogenic risk factors; in addition, they point to the need of setting up studies specifically designed to unravel the different biology underlying renal dysfunction in men and women.

Epidemiological cross-sectional associations can offer only speculation about the biology underlying them. On one hand we can speculate that, through its pro-inflammatory effect [27,28], resistin may well be deleterious on kidney function, quite similarly to what is believed for cardiovascular disease [19,29,30]. On the other hand, although we have previously shown that serum resistin is inversely associated with eGFR also in relatively young, non diabetic subjects with normal kidney function [17], we cannot exclude that high resistin is simply a consequence of reduced glomerular filtration rate, a hallmark of aged diabetic patients as those we here studied. Under this scenario, resistin would be an important mediator of the deleterious effect of reduced kidney function on the risk of cardiovascular disease.

Strengths of our study are the overall sample size, consisting of a total of 1,560 diabetic patients and the fact that the resistin measurements were centralized. In this context, the observed difference in serum resistin concentration between the two cross-sectional studies, with Boston participants having 20–30% lower mean levels as compared to SGR individuals, is somewhat surprising. One possibility is that such difference was due to the different proportion of patients treated with lipid lowering agents in the two studies (76% in the Boston sample as compared to 33% in the SGR sample). Such agents are mainly statins, which are known to reduce resistin concentrations [31,32]. This hypothesis is supported by the observation that participants in the SGR sample who were on statins treatment had serum resistin levels 10% lower than patients who were not on statins (data not shown). Another possibility is that in the Boston sample serum resistin is lower because in some cases it has been measured in non fasting status, a condition reported to decrease its circulating concentration [33]. At variance, given that several studies have reported that circulating resistin levels are relatively stable within individuals over time, we can exclude that the observed difference in serum resistin levels between SGR and Boston samples is due to the different storage time [34–36]. Regardless of the reasons for this discrepancy, the fact that the inverse association between resistin and eGFR is fully replicable across the two studies makes our finding especially convincing.

While a previous report have shown that the association between resistin and GFR remains significant when also C-Reactive Protein (CRP) is taken into account [15], a clear limitation of our study is the lack of high sensitive CRP measurements in both study samples. Such data would have allowed speculations on whether or not the association we observed was mediated by inflammatory pathways. Also the lack of information on different classes of anti- hypertensive and anti- hyperglycemic drugs has to be recognized as a limitation.

A further limitation is the quite clear differences in baseline clinical features between the two samples, which may have played the role in determining some heterogeneity in the association between resistin and kidney function. Being aware of this limitation, we were conservative enough to use random-effect meta-analysis, which takes into account any difference across the two samples. An additional limitation is the very intrinsic nature of our study design, which lacks a prospective observation. In fact, the Boston sample is a cross-sectional observation, while the SGR sample has been followed-up but only for all-cause mortality.

In addition, we cannot say whether or not our present finding is generalizable to populations of different ethnicity with different environmental and/or genetic background, two factors which are possible modulators of serum resistin levels [37].

In conclusion, our data clearly show that there is an independent association between serum resistin and GFR among patients with T2D. Further studies are warranted to examine the exact mechanisms underlying this relationship and to explore the potential role of resistin as a tool for improving prediction, prevention and treatment strategies aimed at reducing the burden of kidney function loss in such high risk individuals.

## Supporting Information

S1 File Table A, Clinical characteristics of patients according to eGFR (≥/<60ml/min/1.73m^2^). Table B, Correlation (r values) between serum resistin concentration (ng/ml) and clinical features.
(DOC)
