# Correction: Serum Resistin and Glomerular Filtration Rate in Patients with Type 2 Diabetes

**DOI:** 10.1371/journal.pone.0127250

**Published:** 2015-04-23

**Authors:** The PLOS ONE Staff

During typesetting, an error was introduced into the first author’s name. The first author’s given name should be: Lorena. The first author’s surname should be: Ortega Moreno.

The correct citation is: Ortega Moreno L, Salvemini L, Mendonca C, Copetti M, De Bonis C, De Cosmo S, et al. (2015) Serum Resistin and Glomerular Filtration Rate in Patients with Type 2 Diabetes. PLoS ONE 10(3): e0119529. doi:10.1371/journal.pone.0119529

The publisher apologizes for the error.
