# Supplementary material for: Serum Resistin and Glomerular Filtration Rate in Patients with Type 2 Diabetes
Source: PLoS One. 2015 Mar 26;10(3):e0119529. doi: 10.1371/journal.pone.0119529 (PMC4374786; doi:10.1371/journal.pone.0119529)
Supplement: S1 File — Table B, Correlation (r values) between serum resistin concentration (ng/ml) and clinical features. (DOC) [file pone.0119529.s001.doc]

Supporting information S1

| Table A in S1 File. Clinical characteristics of patients according to eGFR (≥/<60ml/min/1.73m^2^). | | | | | | |
| --- | --- | --- | --- | --- | --- | --- |
|  | **SGR sample** | |  | **Boston sample** | |  |
|  | **eGFR(≥60ml/min/1.73m^2^) (N=620)** | **eGFR(<60ml/min/1.73m^2^)**  **(N=142)** | **P** | **eGFR(≥60ml/min/1.73m^2^)**  **(N=554)** | **eGFR(<60ml/min/1.73m^2^)**  **(N=244)** | **P** |
| Sex (males %) | 330 (53.2) | 58 (40.8) | 0.14 | 379 (68.4) | 142 (58.2) | 0.22 |
| Age (yrs) | 60.5±9.3 | 68.6±7.9 | <0.001 | 63.4±6.8 | 66.8±6.3 | <0.001 |
| Smokers (%) | 153 (24.7) | 20 (14.1) | 0.03 | 274 (49.5) | 125 (51.2) | 0.84 |
| BMI (kg/m^2^) | 30.9±5.5 | 30.8±5.7 | 0.78 | 32.2±5.7 | 32.4±5.6 | 0.60 |
| Waist circumference (cm) | 101.9±13.2 | 103.3±14.8 | 0.43 | 113.7±12.8 | 113.2±14.0 | 0.60 |
| Diabetes duration (yrs) | 9.8±8.5 | 15.5±9.9 | <0.001 | 11.7±7.2 | 15.5±8.5 | <0.001 |
| HbA1c (%) | 8.6±1.9 | 8.7±1.9 | 0.93 | 7.4±1.3 | 7.4±1.3 | 0.91 |
| Insulin treatment (%) | 227 (36.6) | 91 (64.1) | <0.001 | 224 (40.4) | 141 (57.8) | 0.008 |
| Hypertension (%) | 287 (46.3) | 105 (73.9) | 0.0018 | 388 (70.0) | 208 (85.2) | 0.099 |
| Lipid-lowering therapy (%) | 190 (30.6) | 65 (45.8) | 0.024 | 409 (73.8) | 199 (81.6) | 0.42 |
| Resistin (ng/ml) | 9.8±8.4 | 11.8±7.2 | <0.001 | 6.6±4.2 | 9.5±7.0 | <0.001 |

Continuous variables were reported as mean±SD whereas categorical variables were reported as total frequency and percentages. SGR: San Giovanni Rotondo; BMI: Body Mass Index; HbA1c: glycated haemoglobin.

Table B in S1 File. Correlation (r values) between serum resistin concentration (ng/ml) and clinical features.

|  | **SGR sample (n=762)** | **Boston sample (n=798)** |
| --- | --- | --- |
| Sex (M/F) | -0.03 | 0.10^**^ |
| Age (yrs) | 0.05 | 0.16^**^ |
| Smokers (yes/no) | 0.03 | 0.02 |
| BMI (kg/m^2^) | 0.11^**^ | 0.05 |
| Waist circumference (cm) | 0.15^**^ | 0.06 |
| Diabetes duration (yrs) | 0.03 | 0.09^*^ |
| HbA1c (%) | 0.12^**^ | -0.01 |
| Insulin treatment (yes/no) | 0.09^*^ | 0.07^*^ |
| Hypertension (yes/no) | 0.12^**^ | 0.11^**^ |
| Lipid-lowering therapy (yes/no) | -0.08^*^ | 0.02 |

Correlations between serum resistin and clinical features were analyzed by Spearman correlation. ^*^p-value<0.05; ^**^p-value<0.001. SGR: San Giovanni Rotondo; M/F: male/female; BMI: Body Mass Index; HbA1c: glycated haemoglobin.

Raw data on resistin levels and association with related variables can be provided upon request for collaborative purposes.
